# An Overview of Micro- and Nanoemulsions as Vehicles for Essential Oils: Formulation, Preparation and Stability

**DOI:** 10.3390/nano10010135

**Published:** 2020-01-12

**Authors:** Lucia Pavoni, Diego Romano Perinelli, Giulia Bonacucina, Marco Cespi, Giovanni Filippo Palmieri

**Affiliations:** School of Pharmacy, University of Camerino, 62032 Camerino, Italy; lucia.pavoni@unicam.it (L.P.); diego.perinelli@unicam.it (D.R.P.); giulia.bonacucina@unicam.it (G.B.); gianfilippo.palmieri@unicam.it (G.F.P.)

**Keywords:** essential oil, nanoemulsion, microemulsion, surfactant, co-surfactant

## Abstract

The interest around essential oils is constantly increasing thanks to their biological properties exploitable in several fields, from pharmaceuticals to food and agriculture. However, their widespread use and marketing are still restricted due to their poor physico-chemical properties; i.e., high volatility, thermal decomposition, low water solubility, and stability issues. At the moment, the most suitable approach to overcome such limitations is based on the development of proper formulation strategies. One of the approaches suggested to achieve this goal is the so-called encapsulation process through the preparation of aqueous nano-dispersions. Among them, micro- and nanoemulsions are the most studied thanks to the ease of formulation, handling and to their manufacturing costs. In this direction, this review intends to offer an overview of the formulation, preparation and stability parameters of micro- and nanoemulsions. Specifically, recent literature has been examined in order to define the most common practices adopted (materials and fabrication methods), highlighting their suitability and effectiveness. Finally, relevant points related to formulations, such as optimization, characterization, stability and safety, not deeply studied or clarified yet, were discussed.

## 1. Introduction

Essential oils (EOs) are hydrophobic liquids containing a mixture of volatile molecules, obtained from the plants metabolism as byproducts. In particular, they are synthetized by aromatic plants, i.e., Asteraceae, Apiaceae, Lamiaceae, Lauraceae, Myrtaceae, Rutaceae, and Verbenaceae, that belong to the angiosperm family [1]. They are generally stored in secretory glands where they are present as liquid droplets [2]. They can be obtained from different parts of the plants, such as roots, stems, leaves, flowers and extracted through hydrodistillation, steam distillation, dry distillation, or mechanical pressing procedures [3]. 

Although EOs are complex mixtures of around 20–60 low molecular weight compounds (alkaloids, phenolos and terpenes), their bioactivity is mainly dependent on a few molecules contained at higher concentrations [3]. However, the compounds contained at low percentages are useful to enhance the effectiveness [4,5]. Therefore, EOs show a mutual synergistic activity exploiting the multiple complementary mechanisms of action of most of the components. 

Since the Middle Ages, EOs have been widely used in the traditional medicine as antimicrobial, antioxidant, anti-inflammatory, expectorant, digestive, diuretic agents, as well as foodstuffs preservatives and insecticides. At the moment, one of the biggest challenges of modern botany and of part of the scientific community is to move towards the replacement of synthetic compounds with natural ones, including EOs, in order to exploit their biological activities and to promote sustainable development based on a circular economy [6]. In addition, EOs have been recognized as GRAS (Generally Recognized As Safe) substances by both the US FDA (Food and Drug Administration) and the EPA (Environmental Protection Agency). Hence they are generally considered lower hazardous substances and thus able to reduce the risks for the environment, animals and human health [7], even if this aspect has to be better clarified through proper risk evaluation procedures [8]. In this direction, the EOs industry is exponentially growing (5% per year), showing to be capable to endorse their widespread use [9].

Although up to the present the biological properties and the uses of EOs have not changed much, deeper knowledge of their mechanism of action has been gained in order to guarantee a more rational exploitation, mainly in pharmaceutical, cosmetic, agricultural and food fields. For example, the antimicrobial properties can be mainly ascribed to EOs ability to interact with biological membranes, affecting the membrane potential and, thus the permeability, the transport of nutrients and ions [10]. The cytotoxic action is of great importance in the applications of EOs against certain human pathogens as well as for the preservation of food [11]. Recently, medical studies showed a possible anticancer activity of some EOs, attributable to an antimutagenic (i.e., *Salvia officinalis* L., *Origanum onites* L. and *compactum*, *Lavandula angustifolia* EOs [12,13,14,15]) and prooxidant activity (i.e., *Origanum compactum*, *Artemisia herba-alba* and *Cinnamomum camphora*, *Glandora rosmarinifolia* EOs [16,17]). This latter is a fundamental property to reduce local tumor volume and cell proliferation [10]. Moreover, EOs were showed to be effective botanical insecticides, exerting their toxicity mainly through neurological mode of actions. In fact, they act on acetylcholinesterase (i.e., *Zingiber officinale* Roscoe, *Dysphania ambrosioides* L. EOs [18,19]) and gamma-aminobutyric acid (GABA) A (for some EOs specific components as i.e., α-thujone, thymol, menthol [20,21]) receptors and on the octopaminergic systems (for EOs specific components as i.e., thymol, eugenol [22]) [23]. Although in the past EOs were used in the cosmetic field as fragrances, nowadays they demonstrated several other interesting properties. Among the most important, EOs act as emollients, anti-acne, anti-aging and anti-wrinkles, promoting the synthesis of collagen and elastin. Thanks to their anti-inflammatory effects, EOs could be used to treat or prevent skin inflammations as well [24]. 

EOs showed a large number of biological activities, however their practical use is often limited by several drawbacks that could incur during handling or storage. They are susceptible to chemical conversion or degradation reactions, such as oxidation, isomerization, polymerization and rearrangement that are mainly dependent on environmental parameters as temperature, light and atmospheric oxygen [25]. These instability issues may result in a reduction or loss of effectiveness [26]. Moreover, EOs have poor physico-chemical properties, such as water insolubility (being EOs lipophilic compounds), high volatility and quick half-life that make EOs difficult to handle and use [23]. In this direction, nanoencapsulation technology represents one of greatest challenges, but at the same time promises, for the vehiculation of EOs. Thanks to their subcellular size, nanocarriers are able to enhance the bioactivity of EOs since they allow a deeper tissue penetration and an easier cellular uptake. Moreover, they allow control and modulation of the release of active ingredients on the target site [27]. Finally, the nanoencapsulation approach could improve the EOs’ physico-chemical properties and stability, by enabling their water dispersability, reducing their volatility and by protect them from the interaction with the environment [28].

In this scenario, nanotechnology developed different formulations strategies that could be classified according to on their physical state or composition. Specifically, they provided several approaches such as the preparation of polymeric, metal, and metal oxide nanoparticles and lipid carriers, including liposomes and solid lipid nanoparticles, and micro- and nanoemulsions. Among them, micro- and nanoemulsions (MEs and NEs, respectively) are the easiest to formulate and handle and they can be obtained at low cost [29]. They are suitable in presence of lipophilic or low water soluble compounds, such as EOs, that required the dispersion in water media, i.e., pesticides or foodstuff ingredients [30,31]. Such delivery systems could enhance the bioavailability, and thus the effectiveness, of active compounds through their solubilisation into small oily droplets [32,33]. 

**Figure 1:**
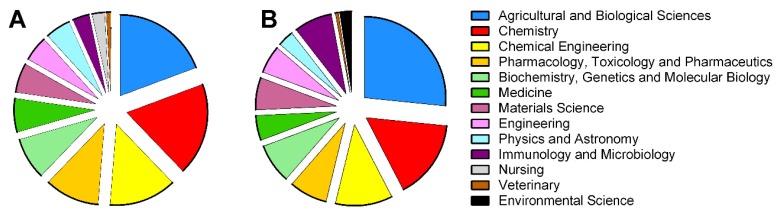
Pie charts of the Scopus database subject area (period 2009–2019) related to scientific production of the essential oils based microemulsions (**A**) and nanoemulsions (**B**).

The interest in EO-based MEs and NEs is increasing as testified by the growing number of studies dealing with them [29]. In this respect, the main fields of applications can be identified through the Scopus database subject area analysis (Figure 1). The top three subject areas, Agricultural and Biological Sciences, Chemistry and Chemical Engineering, cover almost 50% of scientific production. Interestingly, the most represented subject area (Agricultural and Biological Sciences), includes the categories related to agriculture, animal and insect science as well as food science. 

This work aims to offer a technical overview on the formulation, preparation and stability parameters of MEs and NEs when used as vehicle and delivery systems for EOs, based on the most relevant works reported in the literature in the last 10 years. Moreover, it aims to provide a formulation guide for researchers that need to encapsulate EOs in such nanosystems.

## 2. Micro- and Nanoemulsions: Classification

MEs and NEs can be defined as the nanometric dispersion of one liquid phase into another one in which it is not soluble. They are generally composed of a water and an oil phase; their dispersion of one in the other is allowed by the presence of amphiphilic molecules able to decrease the interfacial tension between the two immiscible phases, called surfactants [34].

Although they show many similarities, MEs and NEs also possess significant differences that allow their identification and classification [35]. 

However, over the years some authors described them indiscriminately, generating confusion and misinterpretations about the nature of these two colloidal systems. For this reason, in this section we will report univocal parameters for the definition MEs and NEs, providing identification guidelines following the classification reported by McClements [36]. One of the main issues of misunderstanding is due to the terminology. In fact, the prefixes “micro-” and “nano-” used to designate them let us understand a difference related to the particle size of the dispersed phase. The first one means 10^−6^, indicating micrometric dimension, while the second one means 10^−9^, indicating the nanometric range. However, despite the different terminology, the size distribution of MEs and NEs oil droplets can be overlapped, having values in the nanometric range. In addition, also the definition of nanometric range is not univocally reported. In fact, several authors defined different critical size values, with the upper limit fixed at 100 nm, 200 nm or 500 nm [37,38,39]. 

The main parameter that allows us to discriminate MEs and NEs is the free energy of the system, which influences their preparation process and stability. ME is a thermodynamically stable system whereas NE is not thermodynamically but rather a kinetically stable one [36,40]. This aspect depends on the different free energy of the colloidal dispersion with respect to that of the two separated phases. MEs are energetically favoured with respect to the separated phases, thus they can be achieved spontaneously, by mixing water, oil and surfactant. Nevertheless, an external energy, i.e., heating or magnetic stirring, is often provided in order to overcome the kinetic barriers that could retard the formation of the MEs.

On the other hand, in the case of NEs, the separated phases possess a lower free energy respect to the colloidal system. For this reason, NEs formation is energetically disadvantageous. In this case NEs can be achieved only in the presence of an external energy input that allow to exceed the energy gap between the separated phases and the colloidal system. As a function of how the external energy input is provided, the methods for the preparation of NEs can be divided in high and low-energy ones. This subject will be treated in depth in the Section 4. The different free-energy between these two systems is also a crucial parameter that influences the long-term stability. 

**Figure 2:**
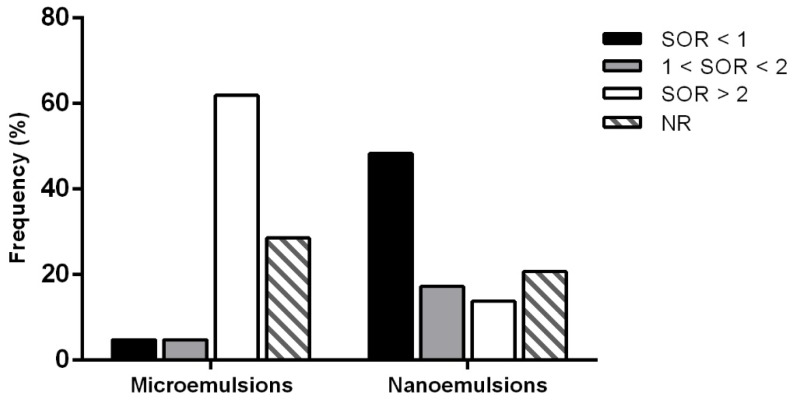
Surfactant-to-oil ratio (SOR) values of essential oils-based micro- and nanoemulsions, expressed as percent frequency, on the basis of the data obtained from the Scopus database (period 2009–2019). The publications that do not contain enough information to calculate it were classified as NR (Not Reported).

Although MEs and NEs require the same ingredients (water, oil and surfactant), they show fundamental differences on their composition, mainly from a quantitative point of view. NEs, contrary to MEs, are able to load higher amount of dispersed phase in presence of smaller amount of surfactant. In this respect, they can be classified based on the surfactant–oil w/w ratio (SOR), that has been reported to be generally >2 in MEs and comprised between 1 and 2 in NEs [41]. Despite these SOR ranges being commonly reported, in the literature can be found also some publications where NEs were obtained with SOR lower than 1 (Figure 2). In this direction, the choice of NEs is preferred since the low amount of surfactant guarantees a better toxicological/safety profile [42]. Moreover, NEs can be formulated with a larger variety of surfactants while MEs required only molecules able to give an ultralow interfacial tension. 

Since the MEs achievement is strictly dependent on a very low interfacial tension and a favourable packaging of surfactant molecules, the composition are determined by the building of a pseuodoternary phase diagram able to determine the optimal SOR [43]. 

The particle structure in MEs and NEs is almost the same: they are composed of an oil core surrounded by a mono- or multilayers of surfactant addressed with the non-polar tails towards the lipophilic nucleus and the polar heads toward the aqueous medium. However, MEs and NEs diverge in the shape of oil droplets. NEs droplets are generally spherical since they are the results of the reduction of the interfacial area as a consequence of the small radius and the high interfacial tension.

By contrast, MEs droplets can be spherical or not spherical, such as cylinder-like, plane-like or sponge-like, because of their very low interfacial tension. The droplets shape depends on the optimal packaging and curvature of the surfactant molecules at the interface oil-water (and consequently by the type and amount of surfactants) and on the amount of oil [44].

## 3. Composition

MEs and NEs do not show any difference in terms of qualitative composition. In fact, both of them are composed of a water phase, an oily phase that, in the case of this review is represented by EOs (active ingredient) alone or in combination with other oils, a surfactant and sometimes a co-surfactant.

Although there are not strict rules for the selection of the components, the properties of the formulations will depend mainly on the choice of surfactant, and eventually co-surfactant, based on the oil phase [45].

In this section we are going to review the most used ingredients reported in the literature in the last few years. 

### 3.1. Surfactant

Surfactants are surface-active molecules that have both a hydrophilic and a lipophilic domain in their molecular structure [46]. Thanks to their amphiphilic nature, surfactants allow the dispersion of two immiscible phases lowering the interfacial tension up to obtain an enough flexible film able to deform around the droplets with the optimal curvature [47]. During the emulsification process they are rapidly absorbed at the oil–water interface and prevent the droplets’ aggregation [48].

Surfactants are usually classified using the hydrophilic–lipophilic balance (HLB) value, an empirical number included in an arbitrary scale ranging from 0 to 20. It takes into account the contribution of both the hydrophilic and lipophilic parts of the molecules. For a non-ionic surfactant, an HLB value of 0 corresponds to a completely lipophilic molecule while a value of 20 corresponds to a completely hydrophilic molecule [49]. Generally, for the formulation of colloidal systems characterized by the dispersion of an oily phase as those reported in this review (EOs dispersed in aqueous phase), the choice falls in intermediate-high HLB surfactants, generally between 11 and 16. They seem to be more suitable with respect to those with too-low or too-high HLB because they are more prone to move at the interface [50]. The physico-chemical properties of MEs and NEs can be influenced by the HLB of the system as a function of the oil phase selected for the formulation. When the HLB value of the surfactant is as close as possible to that required by the oil, the smallest droplets size with narrow distribution can be achieved [51]. Nirmal et al. carried out a systematic study in order to find out the best HLB for the achievement of lemon myrtle and anise myrtle EOs based NEs. The authors prepared different combination of Tween 80 (HLB 15) and Span 80 (HLB 4.3). The smallest droplet size and polydispersity index (PDI, a dimensionless value that describes the broadness of the droplet size distribution curve) were obtained with surfactants systems having an HLB value of 14 and 12 for lemon myrtle and anise myrtle EOs NEs, respectively [52]. 

Surfactants can also be classified based on their electrical charge as (i) non-ionic, (ii) zwitterionic, (iii) cationic or (iv) anionic. The electrical properties of surfactants have a great impact on the formation and stability of a formulation [53]. This aspect influences the stabilizing mechanism of the polar head of the surfactant with the aqueous medium. In fact, while non-ionic surfactant are stabilized by dipole and hydrogen bond interactions with the hydration layer of water and by repulsive forces due to steric hindrance, ionic surfactants are additionally stabilized by the electrostatic interactions [47]. However, non-ionic surfactants represent the first choice because, with respect to ionic ones, they show a safer toxicological profile and are generally accepted even for oral ingestion [54,55]. In addition, these latter demonstrated their suitability in the preparation of MEs and NEs by assuring low surface tension and rapid adsorption kinetics [46,56] and thanks to their ability to generate a steric barrier via the bulky molecular groups that are directed towards the continuous medium [57]. 

**Figure 3:**
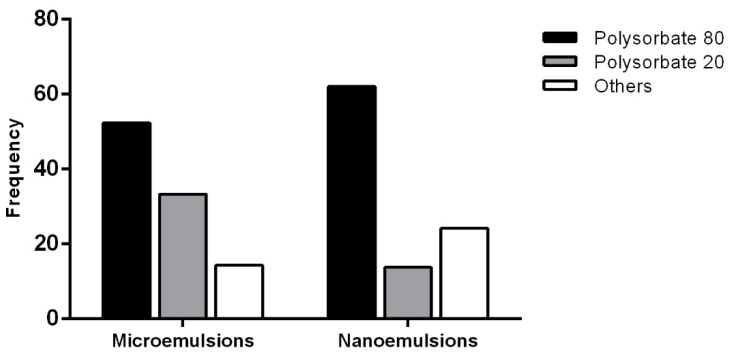
The most used surfactants for the formulation of essential oils-based micro- and nanoemulsions, expressed as percent frequency, on the basis of the data obtained from the Scopus database (period 2009–2019).

At the moment, the most commonly used are: sucrose esters, sorbitan fatty acid esters, glycerol fatty acid esters (polyglycerols), polyoxyethylene sorbitan fatty acid esters (polysorbates) and polyoxyethylene ether surfactants [58]. The two most used surfactants for the formulation of EOs-based colloidal systems are the polysorbates Tween 80 and Tween 20, since they have been demonstrated to give stable formulations without the need of a co-surfactant (Figure 3) [59,60,61,62,63,64]. This is an advantageous aspect both from the formulation and toxicological point of view. In fact, this aspect allows the reduction of the complexity of the phase behaviour and prevents the use of molecules with a poor toxicity profile [65]. Ghosh et al. compared the effectiveness of Tween 20 and Tween 80 on the achievement of eugenol NEs in terms of droplet diameter and stability [66]. Although both of them showed a great impact on droplet size, size distribution and PDI value, Tween 80 was the most effective. In fact, at the SOR 1, NEs with a medium diameter of 190 nm and 95 nm were obtained with Tween 20 and Tween 80, respectively [66]. This could be ascribed to the different structure of the non-polar tail that is saturated and linear for Tween 20 while unsaturated and bended for Tween 80. The presence of double bonds in the hydrocarbon chain was able to give smaller droplets [67] representing an important feature of the surfactants. Li et al. reported the use of Cremophor EL (polyoxyethylene ether surfactants family) in the formulation of finger citron EO. It showed an emulsifying behaviour comparable or even better with respect to that of Tween 80; in fact, it formed a stable interfacial film at a lower concentration. Mixed with the co-surfactants ethanol, 1, 2-propanediol, glycerol, butanol and PEG-400, Cremophor EL was able to give stable NEs along time. Moreover, it enhanced the antibacterial activity of EO [68]. Kim et al. reported the use the non-ionic surfactant sucrose laurate for the formulation of *Ocimum basilicum* EO NE [69]. It was selected for the HLB value (16) and for its good emulsifying properties, thermodynamic stability, biocompatibility and biodegradability [70]. Moreover, it is not toxic, non-sensitizing and non-irritating, thus it can be considered an excellent candidate. Some authors reported also the use of non-ionic surfactants such as Surfynol (2,4,7,9-Tetramethyl-5-decyne-4,7-diol ethoxylate), decaethylene glycol mono-dodecyl ether and polyoxyethylene castor oil derivatives [71,72,73].

In addition to the aforementioned non-ionic surfactants, an interesting alternative is represented by the tri-block polymeric surfactants. Taleb et al. reported the use of Pluronic F127 in the formulation of *Origanum vulgare* EO NE [74]. It is a surface active non-ionic ABA block copolymer having the following structural formula: (ethylene oxide)97(propylene oxide)69(ethylene oxide)97. Although small molecules such as the non-ionic surfactants are able to lower the interfacial tension more than polymeric surfactants, these latter act in a positive manner on another important parameter that occurs in emulsifying process, the interfacial dilational modulus [75]. Therefore, a surfactant mixture of non-ionic and polymeric components could be the optimum for promoting an efficient emulsifying process since an increase in the surface dilational modulus and a decrease in interfacial tension would be observed for the shrinking drops. Due to their lower water solubility, polymeric surfactants are generally stronger absorbed at the O/W interface. For this reason, they desorb with difficult from the interface during the ripening phenomenon, reducing significantly the rate of the instability occurrence [76]. 

In the case of EO-based colloidal dispersions, natural surfactant molecules should represent the first choice. Proteins or polysaccharides have resulted to be the most investigated ones. Nevertheless, they are not suitable in MEs formulation since only small molecule surfactants are able to generate ultralow interfacial tension, which is needed for the formation of such systems. On the contrary, NEs can be achieved using a wide number of surfactants, including proteins or polysaccharides [36]. Although proteins are able to give stable NEs, their effectiveness is reduced by slow absorption during homogenization and by a minor capacity to low the interfacial tension with respect to typical surfactant molecules. Moreover, proteins did not result in being effective either for the production of NEs through low-energy methods as well [56]. Qian et al. carried out a study on the relationship between some different formulation parameters and the mean particle diameter of NEs. Interestingly, they found out that small amphiphilic molecules, such as sodium dodecyl sulphate and Tween 20, were more effective for giving smaller droplets with respect to biopolymers (β-lactoglobulin or sodium caseinate), since they showed different behaviours on the interfacial adsorption [77]. The lower efficiency of proteins or polysaccharides surfactant respect to the synthetic ones was also reported by McClements and Rao [56]. From the other side, positive results were reported by Liang et al. [78] and Majeed et al. [79] by using chemically modified starch as emulsifying agents for the preparation of peppermint and clove EO-based NEs respectively. 

A separate discussion is required for the lecithins [78,79,80,81,82]. They are phospholipids that consists of a glycerol backbone esterified with two fatty acids and a phosphate group, characterized by a zwitterionic nature [83]. The amphiphilic structure of phospholipids is responsible for the excellent emulsifying properties of lecithin, and the negative charges of phosphate groups provide repulsive electrostatic interactions important to the stability of emulsion droplets [46,84,85]. Asensio et al. developed a preservative NE for hake burgers constituted by a 16% oregano EO stabilized with 3% soy lecithin [86]. Some authors also reported the synergistic emulsifying action of lecithins, in EO-based NEs, in presence of other biopolymers such as Arabic gum and sodium caseinate [87,88]. These latter contribute to prevent instability phenomena such as flocculation and coalescence providing repulsive electrostatic and steric interactions [89]. Lecithins were also used in the preparation of MEs, even if they have the great disadvantage to usually require the addition of co-surfactant molecules, in particular medium chain alcohols [58,65]. Co-surfactant should decrease the stability of lecithins crystalline lamellar structures. Moreover they help the spontaneous curvature of the lipid layer [34].

Saponins are another class of compounds belonging to the natural small-molecule surfactants family. Their surface activity is due to their amphiphilic nature with the presence of a lipid-soluble aglycone and water-soluble chains in their structure. The effectiveness of saponins extracted from different natural raw materials, i.e., the bark of *Quillaja saponaria* Molina and *Sapindus mukorossi* pericarp, was evaluated in the formulation of EOs-based NEs exploitable in agri-food field [90,91]. Ozturk et al. compared the behaviour of *Quillaja saponin* and soy lecithin in the formation and stability of NEs at different experimental conditions. With respect to lecithin, saponins were able to give smaller oil droplets at lower concentration. Moreover, NEs stabilized with saponins showed a higher stability over a wide range of pH (3–8), temperatures (30–90 °C) and presence of salts [92]. Given their effectiveness and eco-friendly nature, saponins could represent an alternative to the synthetic surfactants in several applications such as foods, supplements, cosmetics, and personal care products.

Another emerging class of biosurfactants is represented by rhamnolipids. They are glycolipids produced by certain microorganisms through the fermentation processes. The polar head, made of rhamnose units, and a non-polar tail, consisting of a hydrocarbon chain, confers them amphiphilic properties. Rhamnolipids showed emulsifying properties comparable to those of Quillaja saponin, being able to form NEs with suitable droplet diameters (*d* < 150 nm) at low surfactant-to-oil ratios (SOR < 0.1). Moreover, they gave NEs with different oil phases (medium chain triglycerides, long chain triglycerides, and flavour oils), stable in a wide range of pH, temperature and ionic strength conditions [93]. Although there are no studies that proved the use of rhamnolipids in the formulation of EO-based MEs and NEs yet, Haba et al. reported a study about the production of emulsions containing EOs of *Melaleuca alternifolia*, *Cinnamomum verum*, *Origanum compactum* and *Lavandula angustifolia* using rhamnolipids mixtures [94]. The authors demonstrated that rhamnolipids are suitable surfactants for EOs emulsification. In particular, thanks to their surface active properties rhamnolipids can promote the vehiculation of EOs, enhancing their availability and, thus, their bioactivity. These results seem to support the potential use of rhamnolipids in the EO-based MEs and NEs.

### 3.2. Co-Surfactant

A co-surfactant is generally a surface active amphiphilic molecule that, due to the small size of the polar head, is not able to stabilize an emulsion itself. But rather, it is helpful in the MEs and NEs formation because of it supports synergistically the surfactant action. In particular, a co-surfactant is able to further reduce the interfacial tension whilst increasing the fluidity of the hydrocarbon region at the interface, thereby increasing the entropy of the system [95]. Moreover, it allows a higher penetration of the oil between the surfactant tails, since it partitions between the tails of the surfactant chains favouring the optimal curvature of the interfacial film [58].

The use of co-surfactants is common in ME formulation because, although the free energy associated with the formation of MEs is negative, their addition accelerate the preparation process. Moreover, the achievement of NEs with low-energy methods requires the presence of co-surfactants as well [56,96].

The most common co-surfactants are short/medium-chain length alcohols. They are the most appropriate because they are small molecules with an amphiphilic nature, having a hydrocarbon chain and a hydroxyl group. They diffuse rapidly between the oil and water phases, reaching the interface. Such co-surfactants intercalate between surfactants molecules weakening both the polar heads and the hydrocarbon tail interactions. This leads to a more flexible interfacial film able to deform readily around droplets [45]. Due to their partitioning between the two phases, alcohols could also influence the solubility properties of the oil and water phases [65]. Interestingly, the presence of monoterpene alcohols in some EOs, such as geraniol or terpinen-4-ol and α-terpineol in tea tree oil, enhances the formation of MEs, acting as co-surfactants, since they are composed of two distinct polar and non-polar parts that provide the amphiphilic features to the molecules [69,97]. Within this class of co-surfactants, ethanol is the most used in the formulation of EOs-based MEs and NEs because of it induces the formation of systems able to be diluted in the aqueous phase [98]. Sometimes, the presence of co-surfactants could destabilize the system, especially MEs, on dilution since it could partition away from the interface into the continuous phase [58]. However, ethanol, penetrating the surfactant film at the interface, leads to a decrease of the bending moduli of this film, favouring the formation of water-dilutable MEs. Volpe et al. reported the encapsulation of octyl p-methoxycinnamate into a MEs stabilized by glycol mono-dodecyl ether (surfactant) and ethanol (co-surfactant) [72]. Through pseudo-ternary phase diagrams they investigated the optimal surfactant/co-surfactant ratios. After the stability study, the ratio 2:1 resulted the most effective. The same surfactant/co-surfactant ratio (2:1) was also reported by Xu et al. in the formation of a *Cassia* EO ME in the presence of Tween 20 and ethanol [99]. In the case of NEs, Hashem et al. produced a 14% *Pimpinella anisum* EO NE in presence of 3% of Tween 80 and 3% ethanol (ratio 1:1) through a low-energy method [100].

Other short/medium chain length alcohols commonly used are glycerol, propylene glycol, polyethylene glycol derivative, and sorbitol. Their effectiveness could also be related to the solubility enhancement of the head groups in the aqueous phase [56,65,69,101].

Chaiyana et al. investigated the ability of different surfactants (Tween 20, Tween 80, Triton X-100, Triton X-114, or Span 80) and co-surfactants (ethanol, propan-2-ol, glycerin, PG, or PEG-400) to different weight ratio (1:2, 1:1, 2:1, 4:1) to give stable pomelo EO MEs, by the pseudoternary phase diagram construction. Interestingly, the best combination was Tween 20/PEG-400 at the ratio 4:1 since it gave the largest ME region [102]. Cespi et al. encapsulated *Smyrnium olusatrum* EO into a ME composed of 13% of Tween 80 and a co-surfactants mixture of glycerol/ethanol al the ratio 6:1 [103].

Other common co-surfactants are represented by surfactants having different HLB values with respect to the main one. The use of a second surfactant molecule can facilitate the formation and stabilization of MEs and NEs. The use of hydrophilic and a lipophilic surfactants can lead to an additive effect of the two surfactants [104,105,106]. Therefore, the use of a mixed-emulsified system can enhance the formation of small droplets [56]. Mazarei et al. investigated the effect of different mixtures of Tween 80 (hydrophilic surfactant) and Span 80 (lipophilic surfactant) on particle size and long-term stability. They found that the mixture having HLB value of 10 possessed an appreciable stabilizing effect against droplet growth due to Ostwald ripening [107]. This shows that the HLB value of surfactants mixture could be considered as an important factor that controls the occurrence of instability phenomena such as the Ostwald ripening. 

Interestingly, Paim et al. used a multiple approach on the stabilization of *Poiretia latifolia* NE. In fact, they used a surfactant mixture of Tween 80 and Span 80 at the concentration determined through the HLB study. Moreover, the co-solvent propylene glycol was added at the water phase at the ratio 1:2 [108]. Ghaderi et al. compared the co-surfactant activity of the lecithin and cethyl alcohol in a *Thymus daenensis* NE, in the presence of Tween 80. Although both of them showed to be effective against the droplets growth along time, lecithin was more able in stabilizing the droplets size under 200 nm at all the concentrations investigated [109]. Ziani et al. prepared thyme EOs NEs stabilized by Tween 80 in presence of a cationic surfactant (lauric arginate) and of an anionic surfactant (sodium dodecyl sulphate), used to create positively and negatively charged droplets, respectively. They also investigated the influence of electrical characteristics of the oil droplets on the interaction with the anionic surfaces of microorganisms [110].

The surfactant/co-surfactant ratio is a key factor influencing the phase properties, which depends on differences in the packing of surfactant and co-surfactant at the oil/water interface. For this reason it is not possible to establish fixed ratios since they will depend on the selected surfactant, co-surfactant and oil phase. In this respect, to find out the optimal quali-quantitative composition it is usual to carry out a formulation study. The most used screening tool is the pseudo-ternary phase diagram. It is aimed to investigate the concentration ranges of components (surfactant, co-surfactants, oil phase) for identifying the existence of MEs regions since they are one of the different association structures that can be formed (including emulsion, micelles, lamellar, hexagonal, and cubic and various gels and oily dispersion), depending on the chemical composition and concentration of each component [45,69,99,102]. For example, Volpe et al. built pseudo-ternary phase diagrams for the achievement of *Ocimum basilicum* ME by varying the percentages of the aqueous phase, surfactant:co-surfactant mixture (1:2, 2:1 and 3:1 ratios) and oil using the aqueous titration method. The ME region was defined as visual transparent and isotropic regions with respect to the turbidity ones that revealed the formation of different structures. 

In presence of two surfactant, another commonly used method for the selection of the best ratio is the HLB method (Equation (1)) that provides the mass ratio of each surfactant in order to obtain the desired HLB [111].

M_a_ = (HLBd − HLBb)/(HLBa − HLBb) (1)

where M_a_ is the mass ratio of surfactant a, HLBd is the desired HLB value, HLBa and HLBb are the HLB values of surfactant a and b, respectively.

Through this approach Shahavi et al. achieved a stable clove NE at the HLB 9, by mixing 44 wt% of Tween 80 and 56 wt% of Span 80 [112]. 

### 3.3. Oil Phase

In such systems the oil phase could consist in a carrier oil in which the lipophilic bioactive compound is dissolved or, as in the case of the object of this review, in the active ingredient itself, that are EOs. 

The properties of the colloidal system, as well as the formation and the stability, are strongly influenced by the physico-chemical properties of the selected oil, i.e., viscosity, interfacial tension, polarity, density and refractive index [56].

In the NEs formulation, smaller droplets are usually achieved in presence of oils having low viscosity and interfacial tension [113]. Low viscosity oils require a shorter time to be disrupted by the external energy inputs while a lower water/oil interfacial tension makes easier the size reduction process since less energy is required [76]. Moreover, highly hydrophobic oils impede the formulation of NEs through the phase inversion method [114]. For these reasons, EOs, possessing low viscosity, low interfacial tension and high polarity, are the optimal candidates for the achievement of proper NEs [115].

Although such oils are advantageous from the formulation point of view, they are inclined to instability phenomena such as coalescence or Ostwald ripening, one of the principal destabilization mechanisms of NEs [56]. In order to slow down the Ostwald ripening occurrence the addition of highly non-polar and large molar volume substances, such as medium- or long-chain triglycerides (MCT or LCT) or some vegetable oils (corn, sunflower, sesame oils), defined as “ripening inhibitors” is needed [23,116,117,118].Acting as a kinetic barrier and making EOs less water soluble, they are able to positively influence their partitioning between the lipid droplets and the aqueous phase [119,120]. For these reasons, several works in the literature reported the mixing of EOs, acting as active ingredient, and ripening inhibitor oils, in order to guarantee the long-term stability of the system. Liang et al. added MCT to peppermint EOs in the formulation of a stable antimicrobial NE, to prevent the Ostwald ripening phenomenon [78]. The same inhibitor was used by Asensio et al. to retard the ripening effect on an oregano EO-bases NE [86]. MCT also proved to be an optimal vehicle to deliver active substances since it was considered able to increase their bioavailability [121]. Vegetable oils, being food grade, can be an optimal choice as ripening inhibitor for the formulation of edible systems. Ziani et al. mixed thyme EOs and corn oil al the ratio 1:3 for the achievement of NE as an antimicrobial agent in food or beverage products [110].

Although the addition of ripening inhibitors is suitable for the formulation stability, some authors showed that their presence, and thus the relative modification of the oil phase composition, can influence EOs activity. Chang et al. reported how both MCT and corn oil reduces the antimicrobial activity of the thyme EO loaded into NE [122]. Similar results have been shown by Wan et al., where the same ripening inhibitors decreased the antifungal and mycotoxin inhibitory activity of clove EO [123]. 

In both cases, the bioactivity reduction depended on the concentration and the type of inhibitor. In particular, MCT decreases the efficacy of EOs more than corn oil. This effect could be ascribed to a physico-chemical phenomenon. In fact, the presence of ripening inhibitors results in a higher oil–water partition coefficient of EOs, attenuating the effective amount of EOs delivered and consequently their bioactivity.

Some authors reported the use of other oily substances, in addition to EOs, added for different aims other than ripening prevention. Lou et al., in fact, reported the addition of MCT to the oil phase as a carrier oil. Generally, carrier oils are used in combination with EOs in order to dilute them and alter their absorption rate. Moreover, they should prevent easy evaporation of EOs. Since carrier oils are made from fatty portions, they do not evaporate as quickly. Thus, the presence of carrier oils could help to slow down the absorption rate of EOs, allowing longer efficacy [124].

In the case of MEs formulation, low molecular weight oils are preferred. With respect to high molecular weight oils (i.e., triglycerides), they are able to penetrate the interfacial film enhancing the formation of an optimal curvature of the interfacial film [58]. Moreover, being thermodynamically stable systems, MEs do not incur in instability phenomena such as Ostwald ripening; therefore the addition of oils as ripening inhibitors is not required. Nevertheless, Ma et al. added soybean oil in a system composed of water, EOs and Tween 80 [125]. Soybean oil was able to improve the dilutability of EOs-based MEs and it had a great impact on the formation of the system, expanding the regimes of MEs and reducing the droplet size. It contributed to reduce the EOs volatility as well [126]. 

In some cases, the addition of different oils is needed to overcome instability issues related to the physico-chemical properties of EOs. Cespi et al. selected ethyl oleate, a fatty acid ester used for the preparation of parenteral emulsions and MEs, as solvent to prevent the re-crystallization of isofuranodiene, the most abundant compound in *Smyrnium olosatrum* EO, during the ME storage [103,127]. Ethyl oleate was also used in the formulation of *Pimpinella anisum* EO ME. It showed good solvent properties for *P. anisum* EO crystals (the EOs tend to form solid crystals at temperatures slightly lower than ambient conditions). In particular, ethyl oleate-EO 1:3 was selected as the minimum ratio able to avoid recrystallization during storage. The presence of ethyl oleate enhanced also the physico-chemical properties of the ME, changing the size distribution of the ME from bimodal (pure EO) to monomodal (EO + ethyl oleate) [33,128]. The effect of the ethyl oleate can be explained by assuming that it acts as co-surfactants by penetrating the hydrophobic chain region of the surfactant monolayer [129].

### 3.4. Other Components

Along with the presence of water and oil phases, surfactants and co-surfactants, other ingredients could be found in the MEs and NEs systems, generally for stability reasons.

In particular, thickening agents are used in O/W MEs and NEs to match the density of the oil phase with the surrounding aqueous phase. Thus, acting on the effect of gravitational forces, they could retard the occurrence of creaming or sedimentation phenomena [53]. Moreover, texture modifiers are widely used as well. Hydrocolloids, having thickening properties, are able to modify the texture and the rheology of the system [130], leading to an increase of the stability against the gravitational separation, by retarding the droplets movement [55]. Salvia-Trujillo et al. added sodium alginate, a stabilizer agent, in the formulation of antimicrobial lemongrass EO NE [131]. 

Hydroxyethylcellulose was added to a premixed ME, as water-soluble thickening agents, to improve the viscosity in anti-acne basil oil ME. The increased viscosity, from liquid to gel-like system, provided higher stability on ME. Moreover, it was essential to allow the skin attachment, to enhance the accumulation of antibacterial agent to the target area and thus assuring the anti-acne activity [132].

Generally, water-based systems have to contain a preservative agent to avoid the proliferation of microorganisms. In the specific case of EO-based systems, the addition of preservatives is usually unnecessary since EOs are naturally occurring antimicrobials [133]. Interestingly, Chatzidaki et al. exploited EOs-based ME to encapsulate nisin, an antimicrobial agent [134]. Rosemary, thyme, oregano and dittany EOs were selected to enhance the system’s overall antimicrobial activity, through a synergistic effect of nisin and EOs.

## 4. Fabrication Methods

MEs and NEs require different fabrication procedures for a matter of free energy of the systems.

Since MEs formation is energetically favoured, they can be formed spontaneously by mixing the different components. MEs’ achievement is driven by an energetic process based on the Equation (2) [65]:

Δ*G* = γ Δ*A* − *T* Δ*S* (2)

where Δ*G* is the free energy of the ME system, γ is the tension at the interface O/W, ΔA is the variation of the interfacial area, T is the temperature and Δ*S* is the variation of the entropy of the system.

Considering that this process is energetically favoured and it occurs spontaneously, the free energy of the system (Δ*G*) must be negative. The low interfacial tension and the rise of the entropy of the systems that occur in a ME are high enough to counterbalance the growth of Δ*A* given by the increase of the number oily droplets, allowing to obtain a negative Δ*G* value. Nevertheless, it is usual to provide energy (in form of heating or stirring) to overcome the kinetic barrier or to speed up the rearrangement of the surfactant molecules. 

The methods most used to produce MEs are low energy emulsification method and phase inversion temperature (PIT) method.

The first is achieved by three different procedures: dilution under stirring of internal phase (oil + surfactant) with water; dilution of an external phase (water + surfactant) with oil; and mixing together all the components in the final system. Thus the surfactant/s have to be dissolved in the first phase added (the one that is diluted) or in both. Although in the literature all these three approaches are reported, according to the authors’ experience, in the case of EOs MEs, the “water dilution under stirring of internal phase” procedure is the most suitable one. The low-energy emulsification methods are by far, the most commonly used.

The second method is based on a phase inversion at a specific temperature (PIT). Specifically, a water/oil (W/O) emulsion is prepared and then warm up at to PIT, resulting in a phase inversion (oil/water (O/W) emulsion). During the subsequent cooling, under stirring, a decrease in droplets size and interfacial tension occurs, giving rise to an O/W ME. This method is generally used in presence of ethoxylated non-ionic surfactants [58]. An example of the PIT methods application for EOs is reported by Rao and McClements. They achieved stable lemon oil based-MEs raising the temperature systems at 90 °C for 30 min and then cooling them to ambient temperature. The thermal treatment proved to be an effective tool to overcome the kinetic barrier, probably associated with the positive interfacial tension, and allow the formation of MEs [41].

On the contrary, NEs formation needs of an external energy input to exceed Δ*G* value that in a thermodynamically unstable system is always positive. The methods to achieve NEs can be categorized into high-energy and low-energy methods.

Low-energy approaches are often more effective in producing NEs with small droplet sizes, but they are limited in the types of oils and emulsifiers that can be used (i.e., proteins or polysaccharides are not suitable surfactants) and require higher amounts of surfactants. By contrast, high-energy methods are more versatile because they can use a wide selection of oils and emulsifiers [64]. The reduction of droplets size depends on intensity and duration of energy input, as well as on the properties of oil phase and surfactant. For example, small droplets can be achieved with oil phase having low viscosity and/or interfacial tension, such as EOs or flavour oils, with respect to high viscosity oil as MCT or LCT [56].

High-energy approaches are based on a two-step procedure that provides, at first, the formation of an emulsion through a mechanic stirring, characterized by oil droplets size in the range of micron [41,135]. The second step provides the conversion of the emulsion into a NE through the breakage of oil droplets into small ones by using high-energy mechanical devices, such as high-pressure homogenizers, microfluidizers and sonicators [136]. 

High-pressure homogenizers and microfluidizers mainly diverge in the design of the channel (valve) through which the coarse emulsion is forced to flow. Briefly, in both these systems the coarse emulsion is pumped into a chamber and then forced to pass through a narrow valve under high pressure (in the range of 50–200 MPa). The passage through these valves causes disruptive forces able to break down the large oil droplets into smaller ones, generating a NE. The size of the droplets generated by means of high-pressure homogenizers and microfluidizers mainly depends on the applied pressure and/or the number of passes; in particular the size decreases as these two parameters increase as a function of the viscosity ratio of the oil and water phases [119]. Majeed et al. prepared clove EO NEs by passing the coarse emulsion in a high-pressure homogenizer at different pressures, 50, 100, and 150 MPa for 1, 3, 5, 7, 10, 15 and 20 passes in order to investigate their influence on the final properties of the systems. It has been observed that the droplet size decreased gradually from passes 1 to 5 and after 10 passes the reduction was negligible. The same trend was observed for all the pressures investigated. The smallest droplet diameter, around 150 nm, and PDI was achieved at the highest pressure [79]. 

The sonication technique exploits high-intensity ultrasonic waves, generated by a sonicator probe or bath, to create the disruptive forces able to break up the coarse emulsion into a NE. In particular, the mechanical vibration generated by sonicators are able to carry out a cavitational effect [137]. The size of the droplets achieved through this method depends on the exposure time and intensity of ultrasonic waves, the type and the amount of surfactant, and the viscosity of the two phases [138]. Ghosh et al. optimized the process parameters to formulate basil EO NE through sonication. Authors found out a positive correlation between droplets size and emulsification time and surfactant concentration. The smallest droplets (around 29 nm) were achieved at the sonication time of 15 min and at the 1:4 oil-surfactant ratio [139]. It is important to take into account that the intensity of ultrasonic waves could damage the structure of some component of the system, leading to protein denaturation, polysaccharide depolymerization, or lipid oxidation.

Mazarei and Rafati compared the effect of two high-energy methods, sonication and high-pressure homogenization, on the particle size and the stability of *Satureja khuzestanica* EO NE. High-pressure homogenization proved to be more effective in producing smallest droplets. However, after one week, NEs obtained through both the emulsification approaches showed similar particles size distributions. This could be ascribed by the fact that cavitational phenomenon requires more time for the proper rearrangement of the surfactant around the oil droplets [107]. Another interesting study on the comparison on these two methods is that published by Salvia-Trujillo et al. They reported the impact of the production method on the antimicrobial activity of lemongrass EO NE. While microfluidization enhanced the activity of EO loaded into NE respect to the coarse emulsion, sonication compromised its bioactivity. The time and amplitude of the ultrasound fabrication process led to a complete loss of EO antimicrobial action [140].

By contrast, low-energy methods are based on the transformation of W/O emulsion into an O/W NE. The phase inversion can be achieved by modifying the experimental conditions, such as the temperature (phase inversion temperature, PIT) or the composition (phase-inversion composition, PIC and emulsion inversion point, EIP), in order to reach the inversion point where the interfacial tension is so low to allow the formation of fine oil droplets [29]. 

The PIT method is based on the different non-ionic surfactant solubility, by changing the temperature. Briefly, at low temperatures, the non-ionic surfactants are soluble almost exclusively in water phase forming an O/W emulsion, while at certain temperatures (PIT), they have similar solubility in both the oil and water phases. Raising the temperature at values higher than PIT, the interaction water-head groups of surfactants becomes weaker, the solubility in oil increases and the emulsion reverses into a W/O system. The cooling of the system at temperature below the PIT leads to a rapid movement of the surfactant molecules from the oil phase into the aqueous one causing the spontaneous formation of small oil droplets due to the increased interfacial area and interfacial flow generated. In addition to the classic PIT methodology, several modified approaches are reported in the literature. For example, Hashem et al. added a further step at the end of the process represented by centrifugation at 10,000 *g* of the system [100]. 

The PIT of most surfactant-oil-water systems has been reported to be around 90 °C, suggesting that this method is suitable for the achievement of NEs only in the presence of heat stable oils. For this reason, Chuesiang et al. chose this approach to formulate cinnamon EO-based NEs. Cinnamon EO in fact exhibit good thermal stability, being able to remain stable after heating at 200 °C for 60 min [141].

The PIC approach is comparable to that of the PIT. In this case, though, the optimum curvature of the surfactant film is achieved by modifying the systems composition, i.e., ionic strength or pH, rather than the temperature [142]. 

The EIP method diverges from those the PIT and PIC ones because it occurs through a catastrophic-phase inversion, rather than a transitional-phase inversion [143]. It means that it happens by the alteration of the oil-to-water phases without modify the surfactant properties. Generally, this method is based on the addition of water to an initial W/O emulsion. At the exceeding water content, the coalescence rate of water droplets overcomes that of oil droplets, and so phase inversion occurs (from a W/O to an O/W system). The droplet size achieved through this method depends on many factors, such as oil type, surfactant type (small molecules are preferred since they are able to better stabilize both W/O and O/W systems), SOR, initial surfactant location and rate of water addition [56,144]. At the moment, the analysis in the literature does not allow us to establish a correlation between the physico-chemical properties of oils in bulk (i.e., viscosity and interfacial tension) and the particle size achieved using this method. On the contrary, there is a clear relationship between the SOR and the dimensions of oil droplets. Although comparable particle size distributions can be achieved using high-energy methods and EIP, this latter required a higher amount of surfactant (≈SOR >0.1 and SOR >0.7 for high-energy methods and EIP respectively) [145]. However, the EIP method has the great advantage of allowing the NEs preparation at room temperature by using simple stirring equipment rather than expensive homogenization ones.

Finally, we have to highlight the presence in the literature of different cases reporting the preparation of NEs through methods that cannot be classified as the aforementioned ones. In fact, preparation methods that cannot be attributed neither to the use of high-energy devices nor to procedures involving phase inversion processes (low-energy methods) are reported. In this respect, some authors prepared EO-based NEs (with SOR values proper of NEs) by simply mixing the two phases under the action of a high-speed mechanical stirrer [104,105,146]. Interestingly, this approach represents the first step (of the two generally used) of most common high-energy methods, as well as the classic procedure for the emulsion and MEs preparation.

## 5. Stability

The different free energy status of MEs and NEs influences their long-term stability.

MEs, which are thermodynamically stable at specific conditions, will remain kinetically stable indefinitely as long as the initial conditions (i.e., storage temperature and system composition) will not be modified [36]. 

By contrast, the long-term stability of NEs, that are thermodynamically unstable systems, will depend on the height of the energy barriers between the colloidal system and the separated phases. The lower these barriers are, the quicker instability phenomena will occur. Repulsive interactions (hydrodynamic, steric and electrostatic) between droplets are responsible for the achievement of high energy by preventing the contact and their coalescence. Moreover, as the frequency of contact increases, the rate of instability phenomena occurrence increases. Specifically, this depends on gravitational forces, Brownian motion, applied shear and temperature. 

The main instability phenomena that could lead to phase separation are: creaming or sedimentation (gravitational separation), flocculation, coalescence and Oswald ripening [53]. Nevertheless, the nanometric droplets are subjected to Brownian motions able to overcome gravitational separation forces. Moreover, an effective steric stabilization of NEs is able to avoid the close proximity of droplets, and thus, to prevent the occurrence of flocculation and coalescence phenomena [119]. By contrast, NEs are particularly prone to Ostwald ripening. This phenomenon consists in the condensation and aggregation of smaller droplets into bigger ones that are energetically favoured. Ostwald ripening is driven by the molecular diffusion of oil through the continuous phase. The aqueous phase solubility of the oil is the critical parameter that influences the outbreak of this phenomenon In particular, the Kelvin effect shows how the small droplets have higher local oil solubility than larger ones due to the different Laplace pressure [147]. The rate of Ostwald ripening (*ω*) occurrence has been described by the Lifshitz–Slesov–Wagner (LSW) theory (Equation (3)).

(3) $$\omega =\frac{dr^{3}}{dt}=\frac{8}{9}\left[\frac{C\left(\infty \right)\gamma V_{m}D}{\rho RT}\right]t$$

where *C*(∞) is the bulk phase solubility, *γ* is the interfacial tension, *V_m_* is the molar volume of the dispersed phase, *D* is the diffusion coefficient of the dispersed phase in the continuous phase and *ρ* is the density of the dispersed phase. 

In this respect, the use of oils having very low aqueous solubility, as well as the addition of a second dispersed phase component, are preferred because able to reduce the rate of the occurrence of such instability phenomenon. Specifically high non-polar substances, such as medium-, long-chain triglycerides and vegetable oils (i.e., corn oil, sunflower oil), are less susceptible to Ostwald ripening. Given the high water solubility of EOs, the addition of ripening inhibitors in EOs based NEs is always recommended, since they favoured the partitioning of EOs in the oil phase, avoiding their diffusion through the aqueous one [122]. 

Another parameter that influences the rate of this phenomenon is the interfacial tension; in fact to slow down the ripening occurrence it is necessary to reduce the interfacial tension of the systems, although γ should be reduced by several order of magnitude to be actually influent [75]. In this respect, the choice of surfactants that strongly adsorb at the O/W interface (i.e., polymeric surfactants) is favourable. They are able not to desorb during the ripening, retarding the rate of this phenomenon [76]. 

**Figure 4:**
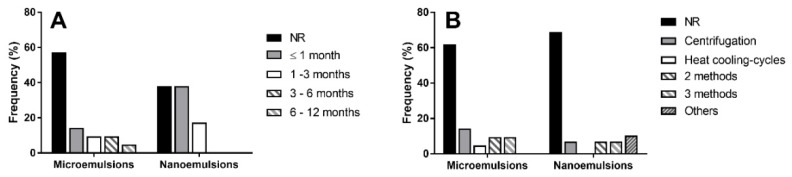
Stability studies (**A**) and accelerated stability studies (**B**) carried out on essential oils-based micro- and nanoemulsions, expressed as percent frequency, on the basis of the data obtained from the Scopus database (period 2009–2019). The publications that do not reported stability information were classified as NR (Not Reported).

Although the stability of these systems is a fundamental aspect, the lack of such information in a remarkable number of publications has to be highlighted. In addition, when such information are present, they are reported only for short periods, such as 30 or 60 days [74,106,111], that cannot be considered sufficient to predict their behaviour over time (Figure 4A). In this respect, some authors used predictive studies about the thermodynamic stability of MEs and NEs. In particular, they evaluated the ability of the systems to keep their features unchanged after different stress conditions such as centrifugation, heating–cooling cycles and freeze-thaw cycles (Figure 4B) [148]. However, according to our experience, the comparison between the real-time stability and the aforementioned predictive methods showed contradictory results (data not published yet). Specifically, it has been observed that, although for some samples instability occurred within 6 months, centrifugation, heating–cooling and freeze–thaw cycles predictive methods did not show any evidence of changing.

## 6. Safety

EOs have been widely used in traditional medicine as well as foodstuff preservatives and insecticides. Their efficacy and biological activity have been broadly evaluated in the last 10–15 years in the different fields of applications by the scientific community [3,134,149,150,151,152]. However, the same can not be stated for the EOs’ safety and toxicological profile, despite the fact that they have been recognized as GRAS (Generally Recognized As Safe) substances by both the US FDA (Food and Drug Administration) and the EPA (Environmental Protection Agency). Nowadays, only few data are available for a limited number of EOs and applications. Specifically, some authors reported toxicological evaluations when EOs are used as botanical pesticides [127,128], while EFSA (European Food Safety Authorities) reported few risk assessments when EOs are used as feed and food additives [152,153]. A more extensive evaluation of the toxicological hazards of EOs is required to define their practical relevance in the different application fields. 

Moreover, when an active ingredient is incorporated in a specific formulation, the risk assessment has to take into account the excipients or additives used for the formulation achievement as well [154]. Specifically, in the case of aqueous nano-dispersions, such as MEs and NEs, the presence of surfactants should be specifically evaluated since they are reported to be potentially hazardous [155,156]. In this respect, NEs are preferred to MEs since the lower amount of surfactant guarantees a better toxicological/safety profile. In addition, in the specific case of nanosystems, it has been recognized that a further risk factor is related to peculiar features (size, surface area, etc…) of the nanomaterial that, according to EFSA “can impart certain changes in properties and biokinetics behaviour, which may also lead to altered toxicological effects compared with corresponding non-nanomaterial” [157,158]. 

Although nanotechnology is proving to be an effective tool for a more promising exploitation of EOs, the scientific uncertainty and deficiency on a reliable assessment of the potential risks of nanosystems on human health and environment represents, nowadays, an important limitation on their widespread application. Increased regulatory oversight is needed to ensure nanomaterials’ appropriate identification, risk assessment evaluation and thus authorization procedures. Along these lines, the European Community is working on the establishment of a clear and comprehensive regulatory framework. EFSA has recently published the guidelines about the security assessment and the authorization procedures in the field of nanosciences and nanotechnologies [157]. This guide is focused on the physico-chemical properties, risk characterization and exposure assessment of nanomaterials, providing practical suggestions on the suitable methods and techniques required for their characterization and authorization. In particular, this document regards nanotechnologies in the food and feed fields, addressing the evaluation of risk to animal and human health, with particular references to new foods, food contact materials, food and feed additives and pesticides. The European Chemicals Agency (ECHA) is also defining the guidelines for nanomaterials risk assessment, but focusing on the environmental risk to support the authorization procedure (four appendices for nanomaterials applicable to Chapters R.6, R.7a, R.7b and R.7c of the IR&CSA guidance) [159]. 

## 7. Final Remarks

Growing interest in the exploitation of the biological properties of EOs is driving the scientific community to the development of suitable formulations for their vehiculation. However, at the moment, most of the scientific publications focus mainly on EOs bioactivity, showing the lack of in-depth investigations about technological aspects of the EO-based formulations. 

In particular, most of the works about EO-based MEs and NEs did not show formulation studies at the bottom of the product development. Several authors refer to fabrication methods and quali-quantitative composition reported previously for synthetic oils without optimizing the procedure for each specific EO. Moreover, EOs have been mainly evaluated in terms of quanti-quantitative compositions, lacking a deep physico-chemical characterization (i.e., viscosity, density, partition coefficient, surface tension). In addition there is a significant lack of long-term stability data that assures the conservation of the physico-chemical features of the systems over time and an almost complete absence of toxicological information on how and if these formulated products impact on the human and animal health and on the environment.

Desirably, the technological research should address an in-depth investigation of possible correlations between the composition of the system and its stability. EO-based MEs and NEs must not only be evaluated for the effectiveness of their bioactivity, but rather also for their physico-chemical properties. This could be achieved through an improvement of the technological knowledge about formulations.

Finally, it is fundamental to highlight that the presence of natural active ingredients, such as EOs, is not sufficient to recognise a product as safe and eco-friendly. Although the green industry rely on EO-based products as promising safe items, we have to take into consideration that most EO-based MEs and NEs contain several ingredients that are not eco-friendly. For example, as mentioned in Section 3.1, the most used surfactant in MEs and NEs formulation are polysorbates, whose safety profile is nowadays a source of debate. In addition, as suggested by EFSA and ECHA, the impact of the effect of nano-encapsulation on EOs’ toxicological profiles should be properly evaluated.
